# Remarriage of men in later life

**DOI:** 10.1093/geroni/igaf122.3306

**Published:** 2025-12-31

**Authors:** Cagri Elmas

**Affiliations:** Akdeniz University, Antalya, Antalya, Turkey

## Abstract

This paper explores the decision-making processes and experiences of men who remarry later in life, focusing on the interplay between remarriage, aging, and masculinity. Unlike first marriages, remarriage in later years involves complex social, economic, and emotional dynamics, making it a critical site for examining how masculine identities and gender roles are renegotiated. Through a explanatory mixed- research methods approach, this research highlights how older men navigate remarriage within the framework of hegemonic masculinity. Findings reveal that while remarriage serves as a means of emotional and practical support, it also functions as a space where men seek to reassert or adapt their masculine identities. The weakening of hegemonic masculine ideals with aging often leads to a renewed emphasis on traditional family structures, reinforcing heteronormative relationships. At the same time, remarried men emphasize the importance of open communication, mutual respect, and emotional connection in fostering fulfilling relationships. By adopting a life course perspective, this study provides a deeper understanding of how gender and masculinity shape marital dynamics in later life. Ultimately, the research contributes to discussions on aging, gender identity, and the evolving nature of masculinity in contemporary society.

